# Recent Approaches to Determine Static and Dynamic Redox State-Related Parameters

**DOI:** 10.3390/antiox11050864

**Published:** 2022-04-28

**Authors:** Cristina Mas-Bargues, Esther García-Domínguez, Consuelo Borrás

**Affiliations:** Freshage Research Group, Department of Physiology, Faculty of Medicine, University of Valencia, Centro de Investigación Biomédica en Red Fragilidad y Envejecimiento Saludable-Instituto de Salud Carlos III (CIBERFES-ISCIII), INCLIVA, 46010 Valencia, Spain; cristina.mas@uv.es (C.M.-B.); esther.garcia-dominguez@uv.es (E.G.-D.)

**Keywords:** oxidative stress, redox state, ROS, seahorse, oroboros, in vivo imaging

## Abstract

Oxidative stress refers to an imbalance between oxidant and antioxidant molecules, which is usually associated with oxidative damage to biomolecules and mitochondrial malfunction. Redox state-related parameters include (1) the direct measurement of ROS, (2) the assessment of the antioxidant defense status, and (3) the analysis of the resulting oxidative damage to molecules. Directly measuring ROS appears to be the preferred method among scientists, but most ROS are extremely unstable and difficult to measure. The processes of determining both the oxidative damage to biomolecules and the antioxidant system status, although both are indirect approaches, provide a reliable method to measure oxidative stress on a given sample. Recently, the Seahorse XF and the Oroboros O2k systems have provided new insights into the redox state from a more dynamic point of view. These techniques assess mitochondrial oxidative phosphorylation function and bioenergetics on isolated mitochondria, cultured cells, or specific tissues such as permeabilized fibers. This review describes a range of methodologies to measure redox state-related parameters, their strengths, and their limitations. In conclusion, all these techniques are valid and none of them can be replaced by another. Indeed, they have the potential to complement each other for a complete evaluation of the redox state of a given sample.

## 1. Oxidative Stress: What to Measure?

Reactive oxygen species (ROS) is an umbrella term that designates a group of molecules derived from molecular oxygen, which are formed by reduction-oxidation (redox) reactions or by electronic excitation [1]. These free radicals are produced endogenously as a natural by-product of the normal cellular metabolism, in particular the mitochondrial metabolism. At low concentrations, some free radicals can exert a physiological role by acting as signaling molecules. However, ROS production can be induced by exogenous stimuli such as UV light, ionizing radiation, diet, and smoking [2], leading to ROS overproduction. Maintenance of redox homeostasis is of utmost importance for proper physiological functions; therefore, cells are equipped with an elaborate antioxidant defense system. An imbalance between ROS production and their detoxification by the antioxidant systems results in oxidative stress. Free radical products that overwhelm antioxidant defenses, in turn, cause oxidative damage to biomolecules, including nucleic acids, lipids, and proteins, thereby affecting cell integrity function. Indeed, oxidative stress has been implicated in a wide variety of diseases [3,4,5].

**Figure 1:**
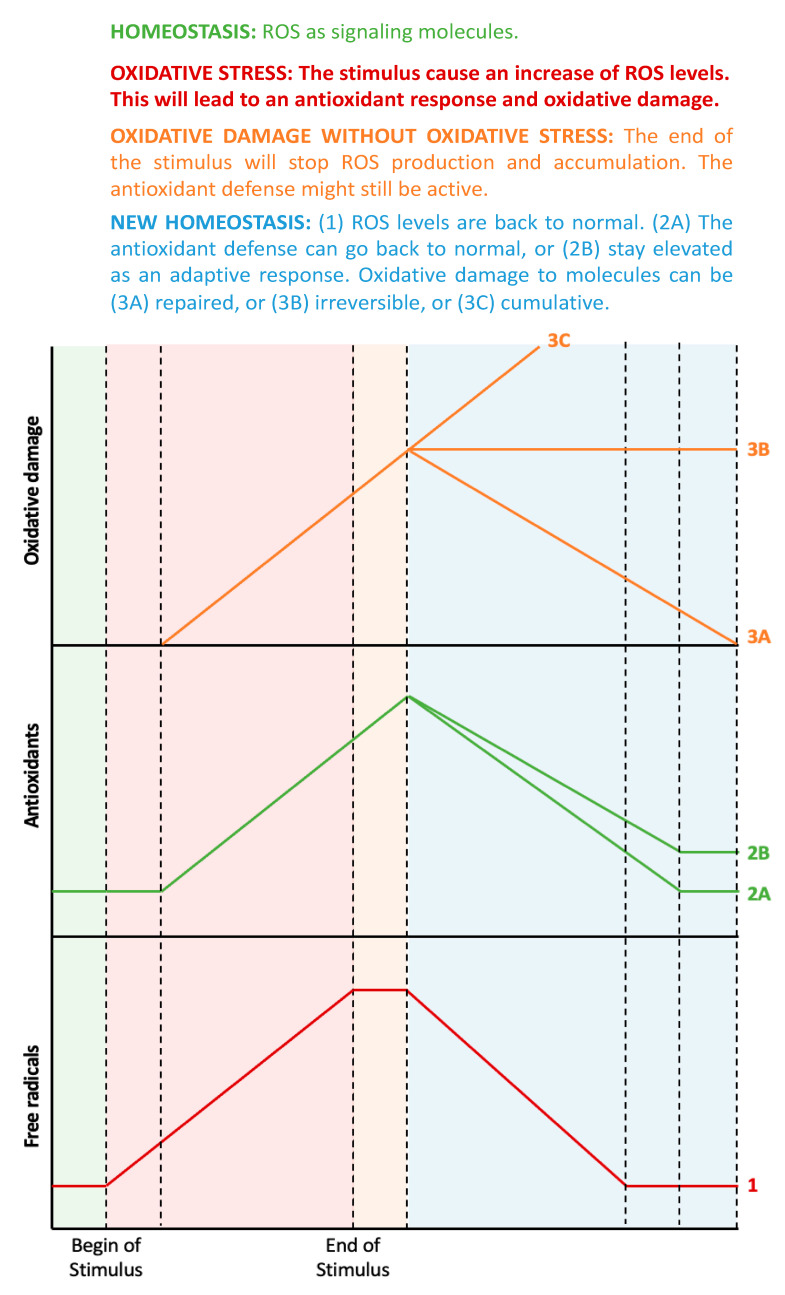
From oxidative stress to oxidative damage: chronological aspects. In homeostatic conditions (the green-shaded area), there is a small amount of ROS that acts as signaling molecules, and the corresponding antioxidant systems maintain ROS at physiological levels. When a prooxidant stimulus appears (pink-shaded area), ROS levels increase, and, therefore, the antioxidant defenses will also increase, but with some delay. During this phase, ROS levels are higher than the antioxidant defenses, thus leading to oxidative stress, which, in turn, will lead to oxidative damage to biomolecules. All these factors (ROS, antioxidant defenses, and oxidative damage to biomolecules) will continue to increase while the stimulus exists. When the stimulus disappears (orange-shaded area), ROS levels will no longer increase, but the remaining ROS molecules will continue to stimulate the antioxidant system and damage molecules. During this phase, there is no more imbalance between ROS and antioxidants, thus, there is no oxidative stress, even though there is still oxidative damage to biomolecules. Finally, the organism aims to return to homeostasis (blue-shaded area), but the antioxidant defenses can decrease to the previous levels or stay elevated as an adaptive response. Similarly, the oxidative damage to biomolecules can be repaired by autophagy or can be accumulative and even irreversible (probably leading to cell death).

The assessment of oxidative stress-related processes can be performed in three ways: (1) through the direct measurement of ROS, (2) by measuring the antioxidant defense status, and (3) by analyzing the resulting oxidative damage to molecules [6]. Figure 1 shows the estimated chronological cascade of events in the oxidative stress-related process.

From a chronological point of view, an initial increase in ROS levels would consequently trigger an increased antioxidant response. Indeed, the imbalance between oxidant and antioxidant molecules, i.e., oxidative stress, would only be detectable during the early stages when the antioxidant response has not yet reached its capacity to fully compensate for oxidative activity. It is important to mention that, at this point, oxidative damage to molecules might not have happened yet, as this is the ultimate consequence of oxidative stress [7]. During later stages (or if the oxidative stimulus persists), ROS levels can still be elevated, but the antioxidant response should also be elevated. In this ideal scenario, both parameters are elevated, with no current oxidative stress. If the increase in ROS has been fully compensated for by the concomitant antioxidative potential, then any molecular damage should be minor. In the case where an increase in ROS levels is observed together with a reduced antioxidant capacity, detectable oxidative damage to biomolecules will be apparent. The main cause of this would be an impaired or exhausted antioxidant response.

Ideally, the assessment of redox balance should include measurements of antioxidants, ROS production, and oxidative damage to biomolecules. Recently, the development of new technologies has enabled the measurement of mitochondrial respiration in vivo, thereby providing a more dynamic approach. This review aims to analyze all the aforementioned parameters, their strengths, and limitations, to provide a more robust assessment of oxidative stress-related processes.

### 1.1. Direct Measurement of ROS

Major ROS that can be measured include the superoxide radical (·O_2_^−^), hydrogen peroxide (H_2_O_2_), singlet oxygen, hydroxyl radical (·OH), and peroxyl radicals (·ROO^−^). Nitric oxide (·NO) and peroxynitrite (ONOO^−^) interact with ROS, and altogether, they form a group of species called RONS (reactive oxygen and nitrogen species). In physiological conditions, a major source of ROS production is the mitochondria; however, other cellular organelles or compartments can also produce ROS, such as peroxisomes, the endoplasmic reticulum (ER), and the plasma membrane. These compartments contain oxidative enzymes such as NADPH oxidases (in peroxisomes and ER) and xanthine oxidases (in peroxisomes). Also, other enzymes, including nitric oxide synthases, lipoxygenases, cyclooxygenases, monooxygenases, and myeloperoxidases, found in different intracellular spaces can produce ROS [8].

The most common way to estimate ROS intracellular levels is using a fluorescence methodology associated with suitable probes [9]. Some probes detect total cellular ROS levels, and some probes can be mitochondria specific. Thus, the measurement of non-mitochondrial ROS can be performed by determining the difference between total and mitochondrial ROS levels. Another possibility is to block the mitochondria before measuring the total ROS production. Also, imaging probes are helpful to confirm locations.

ROS determination is, however, hampered due to their extremely short lifespan; peroxyl radicals and hydrogen peroxide are relatively stable molecules with half-lives of seconds to minutes, but hydroxyl radicals are very reactive and their half-life is limited to nanoseconds (Table 1) [10].

There are also limitations to fluorescence probes which can be attributed to their lack of specificity and sensitivity and the difficulty associated with quantification and data interpretation [11,12]. Indeed, according to Forman et al., measuring ROS with fluorescent dyes is an inappropriate methodology. Reporter dyes are very unspecific, such as in the case of the 1,3-diphenylisobenzofuran (DPBF) probe, which has been used to detect singlet oxygen, hydroxyl radicals, and H_2_O_2_ [13]. Therefore, the proper identification, separation, and quantification of the specific oxidation product are required, in addition to the performing of appropriate controls using inhibitors [14].

Despite being the most widely used probes, 2,7-dichlorodihydrofluorescein diacetate (DCFH-DA) and dihydrorhodamine-123 (DHR123) cannot provide a reliable measurement when it comes to intracellular H_2_O_2_ because of their lack of specificity; i.e., DCFH-DA reacts with hydroxyl radical (·OH) and alkoxyl radicals (·RO^−^) and DHR123 reacts with horseradish peroxidase (HRP) and with hypochlorous acid (HOCl) [15,16]. However, DCFH-DA is a suitable redox indicator of intracellular changes in iron signaling or peroxynitrite formation [15], and DHR123 can be used as a nonspecific indicator of intracellular ONOO^−^ and HOCl formation [17]. The detection of extracellular H_2_O_2_ can be performed using the AmplexRed probe, which is highly oxidized by horseradish peroxidase (HRP) and H_2_O_2_ to the fluorescent resorufin [18]. The advantage of this probe relies on its specificity to H_2_O_2_, but it is tissue unspecific and resorufin is light sensitive.

Mitochondria-targeted hydroethidine (MitoHE) and MitoSox react with superoxide radicals, giving rise to several red-fluorescent products with overlapping spectra. Therefore, these probes are not a reliable indicator of mitochondrial superoxide radical formation [19]. To overcome this spectral overlap limitation, ethidium and ethidine can be separated by HPLC or LC-MS/MS, which in turn increases the specificity for free radicals [20].

Another interesting approach is to monitor the heterogeneous cellular redox state in a spatiotemporal manner. The cellular redox state is governed by pyridine nucleotides (NADPH/NADP^+^ and NADH/NAD^+^), thiols (glutathione and thioredoxin systems), and ROS. Genetically encoded fluorescent sensors can be applied to target specific organelles using specific amino acid extensions. Some of these probes are yellow fluorescent protein (rxYFP)-based redox biosensors, such as iNap (to monitor NADPH/NAD^+^), SoNar and Frex (to assess NADH/NAD^+^), and Hyper (to detect H_2_O_2_). Other probes are based on the green fluorescent proteins (roGFP) to target thiol groups (such as glutathione and thioredoxin systems) [21]. Indeed, Hyper probes have been used to track H_2_O_2_ changes in mitochondria and peroxisomes [22]. Similarly, roGFP sensors have been targeted to various positions of the cytosol, thereby revealing the heterogeneity of glutathione [23]. Taken together, these probes offer a dynamic approach to assess the different biological processes in which pyridine nucleotides, thiol redox systems, and H_2_O_2_ are involved. However, the main drawback of these probes is the overlap of their excitation/emission spectra and their pH-dependency [24].

One step beyond is the in vivo imaging system (IVIS) spectrum, where optical imaging enables the study of molecular mechanisms in living animals using bioluminescent or fluorescent reporters in a non-invasive manner. Bioluminescence imaging (BLI) can be used to detect and monitor biological targets, whereas fluorescence imaging (FLI) is indicated for monitoring and quantifying the cell behavior of biological targets. Therefore, ROS can be detected using BLI. A bioluminescence probe, namely L-012 (an analog of luminol), was initially described for measuring the production of superoxide anion [25]. Subsequent studies revealed that L-012 is nonspecific and can be used to detect other free radicals, such as reactive nitrogen species, and monitor NADPH oxidase activity [26,27,28]. Another more chemoselective bioluminescent probe is Peroxy Caged Luciferin-1 (PCL-1), which is a boronic luciferin molecule that selectively reacts with H_2_O_2_ to release bioluminescent luciferase. This probe has been used for the real-time detection of basal endogenous levels of H_2_O_2_ in mice [29]. Taken together, bioluminescent probes can be sensitive enough to detect H_2_O_2_ in major organ systems such as the brain, the liver, the bladder, or the heart, thus giving rise to the possibility of monitoring several diseases, including neurodegenerative diseases, cirrhosis, and heart disease.

### 1.2. Products of Oxidative Damage

Products of oxidative damage mainly include protein oxidation, lipid peroxidation, and DNA damage, which can be measured in vitro (monolayer cell cultures) and ex vivo (tissues and organs). The following paragraphs describe the most used and accepted methods and technologies for the determination of each type of oxidative damage to products. These methods are summarized in Table 2 and described in more detail in the following subsections.

Focusing on future research, it would be essential to develop a common database to share the different measurements obtained within the scientific community to establish a value range for each parameter. Indeed, we previously published all the MDA measurement values that we have performed in our laboratory using the HPLC methodology [30]. More precisely, we published the range values for MDA in plasma samples from humans under different conditions, such as young/old individuals or healthy/non-healthy subjects. Interestingly, we noticed that these values were not comparable with other species and even between different strains of the same species. Therefore, Table 3 suggests the minimum information that should be mentioned for each parameter when reported.

#### 1.2.1. Markers of Protein Oxidation

When proteins are attacked by ROS, three main modifications may occur hydrogen atom abstraction from C–H, S–H, N–H, or O–H bonds; electron abstraction from electron-rich sites; and the addition of electrons to electron-rich centers [31]. These modifications entail conformational and structural alterations; therefore, a complete analysis of protein oxidation should include the gross modifications of parent proteins, the detection of protein oxidation intermediates, and the detection of end products [32].

One study analyzed parent protein conformational changes subjected to increased oxidative stress levels using size exclusion chromatography (SEC). The results show that SEC elutions were a mixture of protein aggregates and fragments, which were later identified using LC/MS [33]. Other studies have used immunoblotting (such as the western blot (WB) and enzyme-linked immunosorbent assay (ELISA)) techniques with specific antibodies to detect protein structural modifications in Huntington’s disease [34,35] and Multiple Sclerosis [36]. However, detecting minor changes in parent proteins against a large background of unaltered proteins can be very challenging.

Protein oxidation intermediates include radicals, hydroperoxides, chloramines, bromamines, and sulfenic acids. These molecules are transient and often present at low concentrations. Nonetheless, they can be determined by electron spin resonance spectroscopy. Indeed, using this methodology, some peptide-derived radicals have been detected following hydroxyl radical attack on amyloid-β (1–40) and α-synuclein to unravel the protein conformational disorders observed in Alzheimer’s disease and Parkinson’s disease, respectively [37].

The detection of stable protein products such as sulfur-containing amino acids, the oxidation of aromatic amino acids, and protein carbonyls can yield higher quality data and enable quantification. Protein carbonyls can be quantified via their reaction with 2,4-dinitrophenylhydrazine (DNPH) to provide the hydrazone, which is then measured by optical absorbance or by antibodies against standards [38,39,40]. Indeed, oxyblot analysis has been used to detect protein carbonylation in senescent fibroblasts treated with H_2_O_2_ [41]. Moreover, protein carbonyls are very stable and can therefore be considered biomarkers in oxidative stress-related diseases. However, the biological and clinical relevance of protein oxidation as a biomarker is only useful if the employed methodology can identify and quantify the specific modification. MS is probably the most accurate technique since it provides both conformational and quantitative information. Indeed, it is critical to correlate the observed modification of the protein with the observed biological and functional defects and to assess the relation of causality.

#### 1.2.2. Markers of Lipid Peroxidation

Lipid peroxidation can be described generally as a process under which oxidants such as free radicals or nonradical species attack lipids containing carbon-carbon double bond(s). During ROS attack, hydrogen is abstracted from carbon with oxygen insertion, resulting in the formation of lipid peroxyl radicals and hydroperoxides, which are short-lived molecules. Once lipid peroxidation is initiated, a propagation of chain reactions will take place until termination products are produced. The final products can be formed depending on the original fatty acid chain attacked and the number of oxidation events, leading to a complex variety of products [42].

Lipid peroxyl radicals are the first products of lipid peroxidation and are essentially unstable. A recent study developed a fluorogenic antioxidant probe bearing a BODIPY reporter chromophore associated with a-tocopherol as a trap segment that detects lipid peroxyl radicals [43]. However, this study did not specify the particular radical that was being measured, which can only be measured through electron spin resonance (ESR) [44]. Similarly, lipid hydroperoxides are also unstable molecules and can be detected and identified by HPLC [45,46].

Many aldehydes are produced during lipid peroxidation, although the most widely studied is malondialdehyde (MDA), owing to the superficial simplicity of the assay with thiobarbituric acid (TBARS assay). However, in complex biological samples, TBARS reacts with many oxidation products and not only with MDA [14]. Therefore, many derivatization methods have been developed for the specific analysis of MDA by HPLC with visible (HPLC-UV/Vis) or fluorescence (HPLC-FL) detection [30] and mass spectrometry coupled with gas/liquid chromatography (GC-MS, GC-MS/MS, or LC-MS/MS) [30,47,48].

Another common aldehyde used as a marker of lipid peroxidation is HNE. Classical methods for HNE determination have mainly been based on various spectrophotometric and/or chromatographic approaches [47]. Other methodologies include immunochemical techniques. On the one hand, immunohistochemistry and immunocytochemistry are qualitative, morphological techniques that allow for the visualization of HNE within cells (immunocytochemistry) or in tissues (immunohistochemistry) [48]. On the other hand, immunoblotting (such as WB and ELISA) assays offer a more quantitative approach to detect HNE-protein adducts [49]. However, the antibodies used in ELISA are not specific for HNE, and, therefore, a large-scale MS methodology must be performed for HNE detection [50].

Lastly, F2-Isoprostanes are chemically and metabolically stable and nonreactive and are considered the best indicators of nonenzymatic lipid peroxidation. Once again, measurement with ELISA is nonspecific, but several methods have been developed to specifically measure F2-Isoprostanes including LC-MS and GC–MS [51]. Indeed, a study demonstrated that both HNE and F2-Isoprostanes measured by HPLC/MS perfectly correlate with inflammation and oxidative stress in renal disease [52].

When analyzing lipid peroxidation, the original lipidic profile of the sample must be taken into consideration. The oxidation of plasma happens preferentially to that of polyunsaturated fatty acids (PUFAs), and once these are depleted, the oxidation of cholesterol proceeds. Moreover, the composition of PUFAs in the blood is dependent on diet, which hampers comparisons between subjects. Another challenge associated with lipid peroxidation measurements is that hundreds of products are formed. Each product is produced in a different yield and is metabolized and excreted at different rates, which hampers the identification and quantification of all of them [42]. Despite all these issues, the main problem associated with the use of lipid peroxidation as a biomarker of oxidative stress is that it does not provide information on the in vivo origin or trigger of the lipid peroxidation.

#### 1.2.3. Markers of DNA Oxidation

Under oxidative stress conditions, DNA lesions can be produced directly or indirectly [53]. In the direct lesion, guanine gives an electron to the original radical cation, which is then “chemically” repaired [54]. In turn, the guanine cation undergoes hydration to form 8-oxo-2′-deoxyguanosine (8-oxo-dG). The attack of hydroxyl radical on the DNA (indirect lesion) affects either DNA bases (70%) or deoxyribose moieties (30%). The latter gives rise to single-strand brakes (SSB).

Direct and indirect methods have been developed to measure DNA lesions. Direct methods detect specific DNA lesions, whereas indirect methods usually measure strand breaks, which can be assessed by analyzing DNA repair enzymes that convert lesions into strand breaks.

8-oxo-dG, as well as 5-hydroxy-2′-deoxycytidine (5-HO-dCyd) and 8-oxo-dAdo, can be detected using HPLC coupled to electrochemical detection (HPLC-ECD) [55,56]. Another method consists of the derivatization of the DNA bases to make them volatile enough to be separated and analyzed by GC-MS. This technique enables the detection of all DNA lesions. Nowadays, HPLC-MS/MS is the most used method for the detection of specific DNA lesions [57,58,59,60]. Indeed, some studies have reported a positive correlation between 8-oxo-dG levels measured by HPLC-MS/MS and chronic liver inflammation [61] and chronic kidney disease [62]. Alternative and simpler ways to measure DNA damage include ELISA and immunohistochemical analysis [63,64,65], although these methods can be nonspecific, and results should not be reported only based on this type of technical measurement.

The alkaline elution (AE) and the “Comet” assay are indirect methods commonly used to detect DNA strand brakes [66]. When the DNA is damaged, the cell activates the DNA damage response (DDR), which can also be monitored. This approach targets DNA repair proteins such as tumor suppressor p53 (TP53), γ-Histone 2AX (γ-H2AX), ataxia-telangiectasia related kinase (ATR), ataxia-telangiectasia mutated kinase ATM, X-ray repair cross-complementing protein 1 (XRCC1), human 8-oxoguanine-DNA-glycosylase (hOGG-1), and the xeroderma pigmentosum group D (XPD) helicase, which can be measured by flow cytometry, immunoblot, IHC, or HPLC [67,68,69]. The study performed by Martinet et al. showed a complete analysis of oxidative DNA damage where they analyzed 8-oxo-dG levels by ELISA, DNA strand breaks using the Comet assay, and DNA repair enzymes by immunoblot. They found increased levels of all three markers in human atherosclerotic plaques [70].

### 1.3. Antioxidants

Endogenous oxidative stress can be modulated by the prevention of ROS formation or by the quenching of ROS with antioxidants, which can be divided into enzymatic and nonenzymatic components.

Mitochondrial ROS production is regulated, in part, by the MMP. It has been suggested that small increases in the MMP induce ROS formation, whereas slight decreases can reduce ROS formation. Hence, a mild uncoupling of the mitochondrial ETC may represent the first line of defense against oxidative stress [71]. Mitochondrial uncoupling proteins (UCP), specially UCPs 2 and 3, have been found to reduce ROS production from mitochondria. Indeed, the accumulation of ROS leads to the activation of UCPs, which in turn produces a proton leak within the ETC, thereby creating a negative feedback loop that modulates ROS formation [72].

UCPs can be measured by qPCR, WB, and ELISA, although their activity can be determined by assessing the mitochondrial ETC function, and in particular, the proton leak, using respirometers, such as the Seahorse extracellular flux analyzer and the Oroboros Oxygraph-2k system, which are explained in the following sections.

Enzymatic components refer to antioxidant enzymes such as superoxide dismutase (SOD), catalase (CAT), glutathione peroxidase (GPx), glutathione reductase (GR), and glutathione-S-transferase (GST). SOD enzymes catalyze the conversion of superoxide anion into the potential second messenger hydrogen peroxide, which in turn is detoxified by CAT or GPx [73]. The expression of these enzymes can be determined by qPCR and the protein amount can be qualitatively determined by WB; however, their activities are measured using colorimetric assays. SOD activity can be assayed by monitoring the rate of inhibition concerning the reduction of nitroblue tetrazolium (NBT) [74]. CAT activity can be determined by monitoring the disappearance of H_2_O_2_ at 240 nm [75]. GPx activity can be assayed by measuring the decomposition of H_2_O_2_ and GSH [76], and GR activity can be measured by monitoring NADPH depletion [77]. GST activity can be determined using chlorodinitrobenzene as substrate [78]. A proper study of the antioxidant status must report data on the enzymes’ gene expression and protein levels as well as their activities to evaluate their antioxidant potential or capacity. Accordingly, a study reported all these data to suggest that lifelong soya consumption increases the antioxidant defense system in diabetic rats [79].

Non-enzymatic components include glutathione (GSH/GSSG) and vitamins A, C, and E. Within cells, glutathione exists in both reduced (GSH) and oxidized (GSSG) states. In healthy cells and tissue, more than 90% of the total glutathione pool is in the reduced form (GSH) while less than 10% exists in the disulfide form (GSSG). An increased GSSG/GSH ratio is considered indicative of oxidative stress. GSH levels are measured spectrophotometrically using the DTNB reagent, which reacts with sulfhydryl groups [80,81]. Indeed, it has been demonstrated that GSH levels, GSSG levels, and the GSH/GSSG ratio are significantly altered immediately after intense physical exercise in sedentary adults and return to basal levels after recovery, thus demonstrating that this type of physical exercise induces oxidative stress [82]. However, measurement is hampered due to an underestimation of GSH and an overestimation of GSSG because of the auto-oxidation of GSH. Therefore, fluorometric assays have also been developed to measure glutathione levels using fluorescent probes such as ortho-phthalaldehyde (OPA) and monochlorobimane (MCB) [83]. Glutathione detection using only fluorescent probes is not specific enough, but a combination of fluorescence and HPLC could overcome such spectrophotometric limitations. Indeed, a recent study showed that glutathione-OPA adduct is a valid method for the simultaneous measurement of GSH and GSSG to assess the redox status in biological samples [84].

The levels of vitamin A, retinoids and carotenoids, vitamin C (ascorbic acid), and vitamin E (tocopherols), all-powerful antioxidants, are mostly determined by HPLC [85]. It is important to mention that for a molecule to be considered a ROS scavenger, it would need to overcome all the other potentially reactive molecules in the sample [14]. Thus, increased vitamin levels do not necessarily represent an increased antioxidant status. An exception is vitamin E, which reacts rapidly with lipid hydroperoxyl radicals overcoming the propagation reaction, and can therefore be considered as a marker of lipid peroxidation.

In addition, the total antioxidant capacity (TAC) can also be measured to evaluate the oxidative state of a given sample [86]. The different methods used for the evaluation of the antioxidant capacity are grouped into three distinct categories: spectrometry, electrochemical assays, and chromatography. The spectrometry technique includes several tests, among them the ORAC, HORAC, TRAP, CUPRAC, and FRAP tests, which have been described elsewhere [87]. A unique test that measures the total antioxidant capacity is an attractive idea; this fast and easy method would be very useful in clinical analysis to gain a general idea of the antioxidant status. Nonetheless, assessing TAC is associated with some challenges and limitations. Data reported by the different spectrometric tests do not correlate with each other because each antioxidant molecule reacts differently in each test. These TAC tests are very sensitive to the oxidant insult, but in vivo, several oxidant insults are produced at the same time (such as cancer and tobacco smoke), and the TAC of a sample will be different depending on the nature of the oxidant stimulus [88]. Therefore, TAC tests are a complementary measurement to each specific antioxidant enzyme measurement.

## 2. Recent Dynamic Approaches: Mitochondrial Function, Metabolism, and Energetics-Related Determinations

### 2.1. An Overview of Mitochondrial Respiration

**Figure 2:**
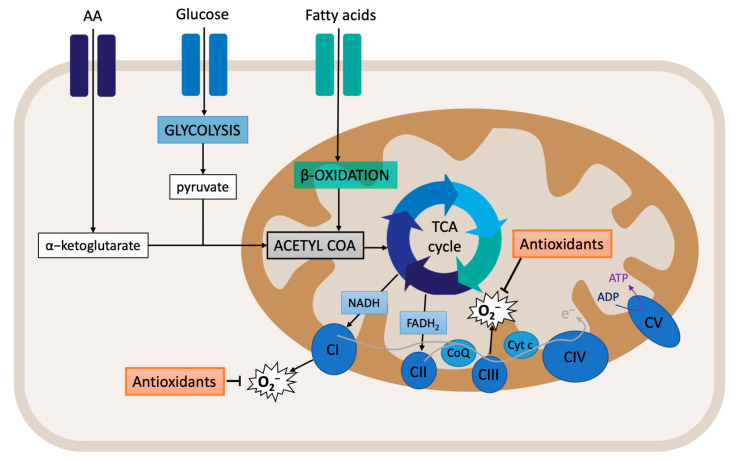
Overview of mitochondrial respiration: oxidative phosphorylation.

Cellular respiration starts with the supply of substrates. Glucose metabolism (glycolysis) produces pyruvate, which is then transformed into acetyl-CoA. Acetyl-CoA is also produced from fatty acid beta-oxidation. Within the mitochondrial matrix, acetyl-CoA enters the TCA cycle and is converted to citrate. At the same time, a-ketoglutarate, which is derived from the hydrolysis of extracellular amino acid glutamine to glutamate, also enters the TCA cycle (Figure 2). The final products of the TCA cycle are the reducing equivalents NADH and FADH_2_, which feed the ETC by transferring electrons to complexes I and II. This process is associated with the translocation of protons across the mitochondrial inner membrane, creating the electrochemical gradient that provides the necessary potential for the ATP synthase (complex V) to produce ATP from ADP and Pi [89,90]. The mitochondrial membrane potential (MMP) is an important component of the electrochemical gradient. An increase in the MMP causes elevated ATP production and reduced electron transport capacities, thus leading to increased ROS production [91]. Indeed, the MMP is critical for maintaining the physiological function of the respiratory chain. The process of oxidative phosphorylation itself is a major source of ROS. Around 1% of the electrons that enter the ETC do not follow the normal flow; instead, they are leaked out of the ETC, and, subsequently, they interact with oxygen to produce ROS [90,92]. Therefore, assessing the MMP and mitochondrial respiration can provide important clues regarding the oxidative stress state of the cell.

The pyridine nucleotides NAD^+^ and NADH provide redox power to the mitochondria for the generation of ATP. These coenzymes exist inside the mitochondria and within the cytoplasm. Although separated, the cytoplasmic and mitochondrial NAD^+^ pools are connected through glycolysis and NAD biosynthesis. Usually, cytoplasmic NAD/NADH ratios range between 60 and 700, whereas mitochondrial NAD/NADH ratios are maintained at 7 to 8 [93,94]. It is known that NAD^+^ levels are limited [95]. Indeed, when excess NADH accumulates, mitochondrial ETC can be overloaded, especially complex I, leading to potential ROS production.

Later, it was shown that complex I reduces NAD^+^ to NADH with electrons received from ubiquinol via reverse electron transfer (RET). Interestingly, RET is known to be associated with the generation of high levels of ROS. RET occurs when the pool of CoQ becomes over-reduced with electrons from respiratory complex II [96]. CoQ can accept and donate electrons, and it can be found in three redox states: (i) oxidized (ubiquinone), semi-oxidized (semiubiquinone), and reduced (ubiquinol). Indeed, the redox state of CoQ indicates where and how electrons leak from the ETC, thereby producing ROS [97,98].

Nearly all of the components of the ETC are sources of ROS production [99]. Indeed, over a dozen mitochondrial sources of O_2_^−^/H_2_O_2_ have been described: complex I (there are two sites: the flavin in the NADH-oxidizing site (site I_F_) and the ubiquinone-reducing site (site I_Q_)); complex II (site II_F_); complex III (site III_Qo_); 2-oxoglutarate dehydrogenase; pyruvate dehydrogenase (PDH); branched-chain 2-oxoacid dehydrogenase; 2-oxoadipate dehydrogenase; glycerol 3-phosphate dehydrogenase; proline dehydrogenase; dihydroorotate dehydrogenase; succinate dehydrogenase; and the electron transferring flavoprotein/ETF: Q oxidoreductase (ETF/ETF: QOR) system of fatty acid β-oxidation [100,101,102,103]. Pioneer studies have located ROS significant production at complexes I and III. Additionally, a significant amount of ROS can also be generated by the TCA cycle enzyme α-ketoglutarate dehydrogenase (α-KGDH) and by monoamine oxidases (MAO) of the outer mitochondrial membrane [104].

The assessment of mitochondrial ETC function was made possible thanks to the Clark-type oxygen electrode, developed in the 1960s, which enabled the polarographic measurement of oxygen consumption rate (OCR) by isolated mitochondria [105]. Oxygen consumption (respirometric OXPHOS analysis) can provide information on damaged mitochondria. This damage is derived from the derangement of the mitochondrial membrane structure and also by defective enzyme systems in membrane transport, dehydrogenases, electron transport, and coupled ADP phosphorylation [106].

This technology was further perfected, and two new apparatus arose: the Seahorse extracellular flux (XF) analyzer and the Oroboros Oxygraph-2k (O2k) system [107,108]. Both of them are respirometers, i.e., they provide a dynamic measurement of metabolic fluxes (rates), in contrast to static determinations (states) of molecular components such as enzyme levels or oxidized biomolecule levels [109].

The Seahorse extracellular flux (XF) analyzer measures oxygen consumption and extracellular acidification in intact monolayer cells in a high throughput multi-well format. The Oroboros (O2k) system provides a High-Resolution FluoRespirometry (HRFR) analysis that combines measurements of oxygen concentration and oxygen flux while monitoring ROS production (H_2_O_2_ and nitric oxide), MMP, ATP production, Ca^2+^, and pH in isolated mitochondria as well as in permeabilized cells and tissues within closed chambers. Both systems can be used with a series of pharmacologic mitochondrial modulators to provide a profile of the ETC function.

### 2.2. Seahorse XF Extracellular Flux Analyzer

Seahorse XF technology uses label-free sensors to detect extracellular changes in these analytes (oxygen and protons) to measure the oxygen consumption rate (OCR) and the extracellular acidification rate (ECAR), respectively. Moreover, the Seahorse XF analyzer runs with several kits to specifically study mitochondrial function, metabolism, and bioenergetics. In the following section, we briefly describe the most used kit to assess mitochondrial respiration, i.e., the Cell Mito Stress Test.

- **Cell Mito Stress Test**

**Figure 3:**
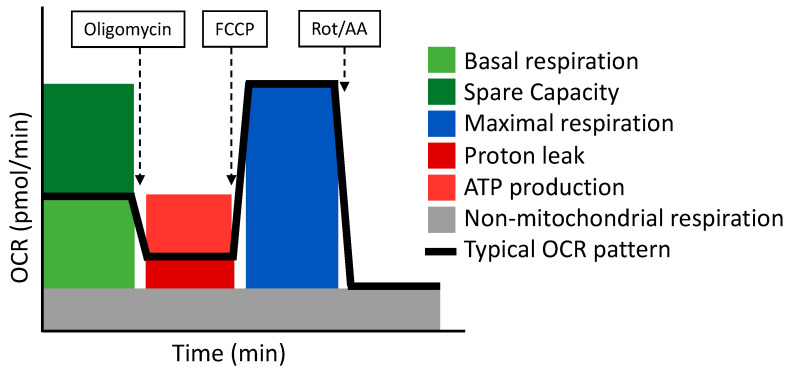
The Cell Mito Stress Test respirometry assay and its related parameters. Adapted from Seahorse (Agilent).

The Cell Mito Stress Test uses three modulators of respiration that inhibit specific components of the ETC. These compounds are oligomycin, FCCP, and a mix of rotenone and antimycin A. Each inhibitor targets a specific component of the ETC (Table 4 and Figure 3). Oligomycin inhibits the ATP synthase (complex V); the decrease observed in OCR following the injection of oligomycin correlates to the mitochondrial respiration associated with cellular ATP production. FCCP is an uncoupling agent that collapses the proton gradient and disrupts the MMP. Consequently, electron flow through the ETC is uninhibited and oxygen consumption reaches its maximum by the entire ETC. The FCCP-stimulated OCR is used to calculate the spare respiratory capacity, defined as the difference between maximal respiration and basal respiration. The mixture of rotenone (a complex I inhibitor) and antimycin A (a complex III inhibitor) stops mitochondrial respiration and enables the calculation of non-mitochondrial respiration driven by processes that are independent of the mitochondria [110].

#### The Cell Mito Stress Test as a Dynamic Approach to Assess the Redox State

Recent findings have proven a correlation between altered mitochondrial function and oxidative stress. Indeed, a study analyzed the beneficial effects of extracellular vesicles (EVs) on oxidative stress-induced senescence of stem cells cultured under hyperoxic conditions. Senescent cells display a high metabolic activity, but when cultured at 21% oxygen, these cells showed high levels of maximal respiration and reserve capacity, suggesting that their metabolism relays on OXPHOS rather than glycolysis. EVs treatment restored the OCR profile towards a more glycolytic metabolism, typical of healthy stem cells [111]. The study performed by Logan et al. analyzed the effect of the decline in insulin growth factor 1 (IGF-1) signaling with age on astrocyte mitochondrial ETC and oxidative phosphorylation. They found that IGF-receptor deficient astrocytes displayed a trend towards a decrease in OCR basal respiration and ATP-linked respiration as well as a reduced OXPHOS coupling efficiency compared to control astrocytes. Moreover, they observed increased ROS production measured by AmplexRed and induction of the antioxidant response [112]. Despite these findings, the authors could not determine the cause or the consequence. Another study aimed to assess the effect of oxidative stress-inducing pesticides (described as risk factors for Parkinson’s disease) on mitochondrial function and ROS production. Their data reveal that upon pesticide treatment, neuroblastoma cells display a decreased OCR at basal and maximal respiration, an inhibited ATP coupling effect, and an increased proton leak [113]. Similarly, a study focused on autistic disorder-derived mitoplasticity. The authors observed that lymphoblastoid cells derived from children with autism have atypical mitochondrial function characterized by an increased reserve capacity at baseline but with an increased vulnerability to acute increases in ROS levels. They interpreted these results as an adaptive resilient response toward chronic exposure to environmental stressors [114]. Consequently, OCR values seem to be lowered in age-related diseases with an important oxidative stress component.

However, this assay has some pitfalls that must be taken into consideration for the correct interpretation of data. Firstly, the basal respiration rate reflects mixed patterns of substrate oxidation. Second, ATP-linked respiration is an ambiguous parameter because it is subjected to complex caveats. Third, the calculation of the maximal respiration and the reserve capacity is based on a non-physiological stimulation with FCCP, which can be applied to many cell types, such as muscle cells subjected to intense exercise. To summarize, the Cell Mito Stress Test is a good starting assay to evaluate mitochondrial respiration; however, some parameters can be ambiguous, hampering their interpretation [115].

Finally, the Seahorse XF analyzer has also some limitations. For all assays, it is of utmost importance to ensure that cells are uniformly seeded in a monolayer and that all wells have the same cellular density [116]. Cell clusters may cause poor cell adhesion and the inaccurate measurement of OCR values [117]. A positive aspect is that the Seahorse XF analyzer automatically injects the drugs through air pressure into each well, but the negative aspect is that only four compounds can be used [118]. However, these drugs are delivered to the media and not to the mitochondria themselves, and, therefore, the permeability of the cell membrane needs to be considered. Moreover, intact cells still lack the in vivo context [119]. Indeed, the in vivo context accounts for a specific oxygen tension. This test lasts for 2 h, and therefore it does not allow the comparison between cells cultured at different oxygen pressures, because, during the assay, all wells are subjected to the same oxygen pressure.

Finally, none of these tests identify the specific site or complex that is altered within the respiratory chain, and therefore a stepwise series of respirometry-based assays are needed to “map” the locations of electron transport deficiency [120]. It is worth mentioning that Oroboros O2k protocols enable the individual study of each complex of the ETC.

### 2.3. Oroboros Oxygraph-2k (O2k) FluoRespirometer

The Oroboros O2k modular system (O2k-Core and O2k-FluoRespirometer) allows for the measurement of mitochondria function using high-resolution respirometry and fluorimetry. The O2k measures the concentration of oxygen dissolved in the aqueous solution inside the experimental chamber. The mitochondrial respiration of the samples is the result of the derivative of the oxygen measurement, so oxygen consumption (oxygen flux) can be obtained. The O2k-FluoRespirometer is the basis for the combined measurement of respiration and fluorometric signals, allowing for the simultaneous monitoring of oxygen consumption together with ROS production, MMP, Ca^2+^, or ATP production.

- **SUIT Protocols**

Specific substrate-uncoupler-inhibitor titration (SUIT) protocols are applied to study different states of coupling control: ROUTINE respiratory activity, respiration coupled to ATP production, excess respiratory capacity, consumption of residual oxygen, and the integrity of cell membranes. Sequential titration of substrates, inhibitors, and uncouplers are applied to assess mitochondrial pathway capabilities in LEAK, OXPHOS, and ET docking control states [121]. Table 5 defines these concepts as well as ROUTINE respiration and ROX.

Like the Seahorse methodology, different segments of the ETC can be interrogated by using specific combinations of substrates and inhibitors. Table 6 describes the recommended combinations to assess each complex of the ETC. The flux-control relationship between non-phosphorylating LEAK respiration and ETC capacity allows for the analysis of intrinsic uncoupling and the experimental uncoupling [109].

**Figure 4:**
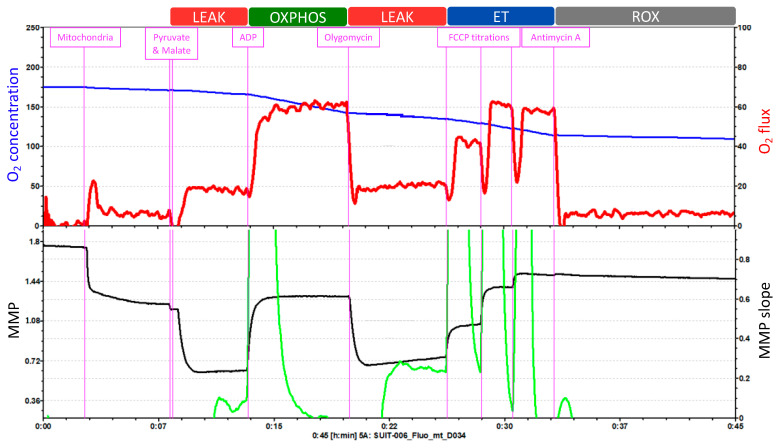
Example of a SUIT protocol pattern on isolated mitochondria. O_2_ concentration and O_2_ flux and MMP measured with TMRM. Adapted from Oroboros O2k.

The Oroboros O2k offers 27 different SUIT protocols that can be applied indifferently to isolated mitochondria, intact or permeabilized cells, permeabilized fibers (from skeletal muscle) or tissue homogenate. The SUIT protocol in Figure 4 consists of five injections that enable the measurement of O_2_ concentration and flux, Ca^2+^, ATP production and pH, together with the assessment of the MMP.

O_2_ concentration is shown in blue (left *Y*-axis) and O_2_ flux is represented in red (right *Y*-axis) as a function of time. The “O_2_ concentration” parameter represents the amount of oxygen within the chamber, and the “O_2_ flux” parameter accounts for the O_2_ consumption.

The first injection is pyruvate followed by malate, to evaluate the LEAK respiration. Because of this injection, O_2_ flux (O_2_ consumption dependent on complex I) increases slightly, which is followed by a hyperpolarization of the mitochondrial membrane. Injection 2 is ADP, to assess the OXPHOS capacity. O_2_ consumption increases and triplicates its previous value; and at the same time, the MMP returns to its normal levels. Injection 3 is oligomycin, which allows for the reevaluation of the LEAK respiration. O_2_ consumption decreases and the accumulation of protons in the intermembrane space by the inhibition of ATP synthase leads again to the hyperpolarization of the MMP. Then, injection 4 is FCCP titrations, to analyze the ET capacity. FCCP elevates respiration and, thereby, O_2_ consumption increases. This uncoupler also depolarizes the mitochondrial membrane in a concentration-dependent manner until the MMP collapse is reached. Finally, antimycin A is injected to evaluate ROX. Antimycin A blocks respiration by inhibiting complex III. Thus, the graph displays low levels of O_2_ flux which represent the residual O_2_ consumption. Moreover, the injection of antimycin A does not further affect the MMP. During the whole length of the assay, the O_2_ concentration (blue line) progressively decreases.

This test cannot measure the integrity of the inner mitochondrial membrane with cytochrome c injection because it would affect the fluorometric measurements of MMP with TMRM. Also, this test does not measure complex IV.

#### SUIT Protocols as a Dynamic Approach to Assess the Redox State

SUIT protocols have been used to assess the role of oxidative stress in the development of many diseases. As an example, the study performed by Wang et al. aimed to analyze whether physical exercise could shift energy metabolism from fatty acid oxidation to glucose oxidation in diabetic rats. The beneficial effect of exercise was supported by an enhanced oxidative phosphorylation level, increased MMP, and a decreased ROS level and oxygen consumption [122]. Similarly, another study evaluated the mitochondrial function and ROS production of circulating blood cells obtained from patients with cardiovascular diseases. They reported consistent data related to reduced mitochondrial respiratory chain oxidative capacity related to the degree of the severity of the disease, as demonstrated by a reduced coupling efficiency and mitochondrial oxygen flux [123].

The mitochondrial respiratory function has also been suggested to be involved in the longevity of each species. Indeed, a study hypothesized that, rather than differences in ROS production, the mitochondrial capacity to consume ROS would be the underlying mechanism to distinguish short and long-lived species. Their results showed similar levels of H_2_O_2_ in the different species tested; however, only long-lived ones showed significantly higher maximal oxygen consumption rates [124]. Since short-lived species are not capable of consuming their own ROS production, antioxidant supplementation appears to be an effective approach to expand lifespan. Indeed, a study assessed the impact of antioxidant supplementation on mitochondrial function of healthy middle-aged men. Their results suggest that antioxidant supplementation mildly suppresses mitochondrial ROS levels without altering mitochondrial function [125]. Supporting the idea that increased mitochondrial ROS levels have harmful effects, a study focused on the impact of mitochondrial oxidative stress within skeletal muscle in a mouse model lacking MnSOD antioxidant defense. They observed that complex II-induced OCR was decreased, and ROS generation was increased in MnSOD deficient mice, leading to severe exercise intolerance [126].

Taken together, these studies support the view that mitochondrial ETC function is useful in assessing the redox state. Importantly, the redox state in the cell exists as a dynamic balance between ROS production and removal. The multiple possible combinations in the addition of substrates, inhibitors, and uncouplers allow for the specific functional assessment of numerous mitochondrial respiratory pathways, including the analysis of minor alterations in respiratory pathway control and/or capacity as well as the detection of coupling defects. The protein analysis of the individual complexes separated by electrophoresis and measured using the western-blotting technique makes it possible to study the structure that is affected in the mitochondria, but it is the fluorespirometer that provides information on the functional status of each of the respiratory chain complexes.

However, SUIT protocols also have some limitations, mainly because they are performed under extreme assay conditions that elicit large effects [115]. This means that the ETC is assayed at maximal capacity, which will only reflect the in vivo situation if the system physiologically reaches this capacity. Therefore, systems that function below their maximum capacity cannot be evaluated. Indeed, SUIT strategies have been developed to specifically analyze the mechanisms underlying OXPHOS but these are unable to correlate OXPHOS with cellular physiology.

Another important limitation of the Oroboros O2k is that the technique is extremely time-consuming. Moreover, a SUIT protocol consists of 10–15 titrations that the researcher must inject manually, making the operator input and labor intensity very high. When using permeabilized fibers, the waiting time between injections is considerably increased, since these samples take longer to stabilize. The Oroboros O2k only allows the comparative analysis of two samples at once, and one is the replicate of the other, and thus the high-throughput capability is reduced. However, a positive aspect of the Oroboros O2k is that results obtained on different days can be analyzed together, whereas Seahorse XF only allows comparisons of samples within the same plate [127].

Despite the limitations, a combination of both Seahorse and Oroboros methods would be the best combination to assess mitochondrial respiration in future studies. Indeed, a recent study used HRFR (Oroboros O2k) to analyze permeabilized and intact platelets and metabolic flux analysis (Seahorse XF) to measure OCR in intact fixed platelets of young and old healthy donors. The results proved that platelets from old donors displayed lower values of oxygen consumption in complex II-linked phosphorylation and complex IV activity, as well as lower resting, uncoupled respiration in both methods [128]. The results obtained by each apparatus correlate with each other; thus, these methods complement each other.

### 2.4. Mitochondrial Bioenergetics Data Interpretation

Experimental interpretations are usually accompanied by the challenges of the experimental manipulation, but also by some assumptions that are made to answer the original hypothesis.

It is commonly assumed that an altered metabolic flux is associated with reduced ATP levels. However, this association is problematic. Indeed, ATP levels are the result of ATP production and hydrolysis; thus, both must be measured [115].

Extracellular flux analyzers compare the relative contributions of OXPHOS and glycolysis with the cell’s energetic state. These parameters can be assayed by measuring the OCR/ECAR ratio. However, this ratio is not quantitatively related to any metabolic flux. Also, the shift from OXPHOS to glycolysis can happen artefactually during the experimental manipulation, thus leading to an incorrect interpretation of the obtained data.

The different samples that can be tested must also be interpreted differently. In this regard, isolated mitochondria/permeabilized cells provide valuable information on the specific biochemical conditions that regulate mitochondrial metabolism, but they lack the physiological in vivo context [129].

Intact cell conditions are more reliable in the physiological context, but the data obtained following their analysis is subjected to ambiguous interpretation due to the complexity of the whole-cell metabolic system.

The mitochondrial metabolism is subjected to functional changes that have often been attributed to a mitochondrial deficiency, dysfunction, or pathological state [130]. However, changes in mitochondrial function are more probably related to the energetic flexibility of the ETC. Indeed, if an altered mitochondrial metabolism triggers a mitochondrial disease or pathology, additional experiments must be performed to support this cause-effect relation.

## 3. Conclusions

Many biomarkers can be measured to assess oxidative stress, such as ROS (using fluorescent probes), oxidative damage to biomolecules (lipids, proteins, and nucleic acids) as well as antioxidant capacity. These direct and indirect approaches can be challenging. Recently, the O2k and the Seahorse XF represent new technological advances that provide dynamic information on the functional properties of mitochondria. Indeed, the gold standard for a proper determination of the oxidative stress-related process of any sample should include the measurement of every parameter. None of these parameters can be substituted for another; rather, they complement each other.

All these techniques have their strengths and limitations, and together they offer an in-depth analysis of the underlying metabolic malfunction in oxidative stress-related diseases.

## Figures and Tables

**Table 1:** RONS and their half-lives.

Species	Half-Life
Superoxide radical (·O_2_^−^)	10^−6^ s
Hydroxyl radical (·OH)	10^−9^ s
Peroxyl radicals (ROO^−^)	7 s
Nitric oxide (·NO)	1–10 s
Hydrogen peroxide (H_2_O_2_)	stable
Singlet oxygen (^1^O_2_)	10^−5^ s
Peroxynitrite (ONOO^−^)	0.05–1 s

**Table 2:** Markers for oxidative damage detection and quantification.

Oxidized Biomolecule	Markers
Protein oxidation	Gross modifications of parent proteins (structural modifications) Protein oxidation intermediates Protein oxidation products (carbonyls)
Lipid peroxidation	Lipid peroxyl radicals and lipid hydroperoxides Aldehydes (malondialdehyde and 4-hydroxynonenal) F2-Isoprostanes Phospholipid peroxidation
DNA oxidation	Specific DNA lesions (8-oxo-deoxyGuanosine) Single strand brakes (alkaline elution or comet assay) DNA damage response system

**Table 3:** Oxidative damage biomarkers: database requirements.

Species and Strain	Condition	Sample Origin	Oxidative Stress Biomarker	Quantitative Methodology	Value Range
Human Mouse Rat Zebrafish	Young/Old WT vs. TG/KD/KO/KI Control/Treated Health/Disease	Blood Urine Cell/Tissue CSF	MDA HNE F2-IsoP 8-oxo-dG	HPLC LC/MS GC/MS MS/MS	[min–max] ± SD

Abbreviations: WT (wild-type), TG (transgenic), KD (knock-down), KI (knock-in).

**Table 4:** Definition of the parameters analyzed in the Cell Mito Stress Test.

Parameter	Definition
Non-mitochondrial respiration	Oxygen consumption that persists after rotenone and antimycin A addition.
Basal respiration	Oxygen consumption used to meet cellular ATP demand and resulting from mitochondrial proton leak.
Maximal respiration	The maximal oxygen consumption rate attained by adding FCCP, which mimics an energy demand by stimulating the respiratory chain to operate at maximum capacity.
Proton leak	Remaining basal respiration not coupled to ATP production.
ATP production	The decrease in oxygen consumption rate upon injection of oligomycin represent the portion of basal respiration that was being used to drive ATP production.
Spare respiratory capacity	The capability of the cell to respond to an energetic demand.

**Table 5:** Definition of coupling states used for mitochondrial energetics characterization.

State	Definition
ROUTINE respiration	Aerobic and anaerobic metabolism is physiologically controlled in the ROUTINE state of cell respiration. Different coupling control states are induced by the application of membrane-permeable inhibitors and uncouplers.
LEAK respiration	After stabilization of ROUTINE respiration, adding oligomycin inhibits ATP synthesis and this resting or unphosphorylated LEAK state is reached, where the LEAK respiration reflects intrinsic uncoupling.
OXPHOS capacity	The OXPHOS capacity is the respiratory capacity of mitochondria in the ADP-activated state of oxidative phosphorylation at saturating concentrations of ADP and inorganic phosphate, oxygen, and defined reduced fuel substrates.
ET capacity	After injecting the membrane uncoupler, the mitochondrial respiratory control via phosphorylation is partially or completely released. Maximal electron transfer (ET) capacity is obtained in the uncoupled open proton circuit state, as the electrochemical backpressure of the CI, CIII, and CIV complex proton pumps are removed to maximally stimulate flow-level respiration.
ROX	Residual oxygen consumption rate (Rox) is obtained after inhibition of the ET pathway in the residual oxygen consumption state (ROX) by sequential titration of complex inhibitors. Various cellular enzymes that consume O_2_ and promote autoxidation reactions give rise to Rox, including peroxidase and oxidase activities that partially contribute to ROS production.

**Table 6:** Specific substrates and inhibitors to assess each mitochondrial pathway.

Complex	Substrates	Inhibitors
Complex I: NADH pathway	Pyruvate + malate or glutamate + malate	Rotenone
Complex II: succinate pathway	Succinate	Malonate
Complex III: ubiquinol cytochrome c reductase		Antimycin A or myxothiazol
Complex IV: cytochrome c oxidase	Tetramethyl-p-phenylenediamine + ascorbate	Azide or cyanide
Complex V: ATP synthase	ADP	Oligomycin
Fatty acid oxidation	Palmitic acid or palmitoylcarnitine or palmitoyl-CoA with carnitine or octanoylcarnitine	Rotenone (Fatty acid oxidation is blocked by inhibition of complex I)
Glycerophosphate dehydrogenase complex	Glycerophosphate	

Note: Electrons flow from NADH to O_2_ with three proton pumps (complex I, complex III, complex IV) in series. Electrons flow from succinate to O_2_ with two proton pumps (complex III, complex IV) in series.

## Data Availability

Not applicable.
